# HHV-6 infections in hospitalized young children of Gabon

**DOI:** 10.1007/s15010-023-02077-w

**Published:** 2023-07-27

**Authors:** Juliana Inoue, David Weber, José Francisco Fernandes, Ayola Akim Adegnika, Selidji Todagbe Agnandji, Bertrand Lell, Peter G. Kremsner, Martin Peter Grobusch, Benjamin Mordmüller, Jana Held

**Affiliations:** 1 Institute of Tropical Medicine, Eberhard Karls University Tübingen, Tübingen, Germany; 2 Centre de Recherches Médicales de Lambaréné (CERMEL), Lambaréné, Gabon; 3 German Center for Infection Research (DZIF), Partner Site Tübingen, Tübingen, Germany; 4 Centre of Tropical Medicine and Travel Medicine, Department of Infectious Diseases, Amsterdam Infection & Immunity, Amsterdam Public Health, Amsterdam University Medical Centres, Location University of Amsterdam, Amsterdam, The Netherlands; 5 Department of Medicine I, Division of Infectious Diseases and Tropical Medicine, Medical University of Vienna, Vienna, Austria; 6 Institute of Infectious Diseases and Molecular Medicine, University of Cape Town, Cape Town, South Africa; 7 Masanga Medical Research Unit, Masanga, Sierra Leone; 8 Department of Medical Microbiology, Radboud University Medical Center, Nijmegen, The Netherlands

**Keywords:** Herpesvirus, HHV-6, Febrile illness, Hospitalized children, PCR, Gabon

## Abstract

**Purpose:**

Fever is a common cause for hospitalization among the pediatric population. The spectrum of causative agents is diverse. Human herpesvirus 6 (HHV-6) is a ubiquitous virus that often causes hospitalization of children in western countries. Previously, we investigated the cause of fever of 600 febrile hospitalized children in Gabon, and in 91 cases the causative pathogen was not determined. In this study, we assessed HHV-6 infection as potential cause of hospitalization in this group.

**Methods:**

Blood samples were assessed for HHV-6 using real-time quantitative PCR. Three groups were investigated: (1) group of interest: 91 hospitalized children with febrile illness without a diagnosed causing pathogen; (2) hospitalized control: 91 age-matched children hospitalized with febrile illness with a potentially disease-causing pathogen identified; both groups were recruited at the Albert Schweitzer Hospital in Lambaréné, Gabon and (3) healthy control: 91 healthy children from the same area.

**Results:**

Samples from 273 children were assessed. Age range was two months to 14 years, median (IQR) age was 36 (12–71) months; 52% were female. HHV-6 was detected in 64% (58/91), 41% (37/91), and 26% (24/91) of the samples from groups 1, 2, and 3, respectively; with statistically significant odds of being infected with HHV-6 in group 1 (OR = 4.62, 95% CI [2.46, 8.90]). Only HHV-6B was detected.

**Conclusions:**

Although tropical diseases account for a large proportion of children's hospitalizations, considering common childhood diseases such as HHV-6 when diagnosing febrile illnesses in pediatric populations in tropical countries is of importance.

**Supplementary Information:**

The online version contains supplementary material available at 10.1007/s15010-023-02077-w.

## Background

In sub-Saharan Africa, infectious diseases are a leading cause of medical attendance among the pediatric population. The causative pathogen is often not routinely diagnosed, and studies revealed that a broad spectrum of pathogens can be responsible for febrile diseases in certain regions [1], whereas in other regions *P. falciparum* malaria remains the leading causative agent for hospital admission [2, 3]. In Lambaréné, Gabon, malaria was the leading cause of hospitalization for fever in children, as shown by a prospective cross-sectional hospital-based study that enrolled 600 hospitalized febrile children and subsequently tried to identify the causative pathogen by advanced laboratory methods [4]. A subset of children remained without a laboratory-confirmed diagnosis when the presence of an initial array of pathogens was assessed. Subsequent analysis for human herpesvirus 6 (HHV-6), cytomegalovirus, and Epstein-Barr virus showed that a relatively large proportion of these children (33%; 29/89), was positive for HHV-6 when analyzed by qPCR. However, it is difficult to fathom whether this was the causative virus for the hospitalization, and the epidemiology of HHV-6 infections in Central Africa is also not well studied.

HHV-6 is a DNA virus that belongs to the Herpesviridae family. It was discovered in 1986 [5] in the peripheral blood mononuclear cells of six individuals with associated lymphoproliferative disorders. Later two variants were identified, HHV-6A and HHV-6B. They differ in genomic aspects as well as epidemiological, biological, and immunological features. Since 2012, they are classified as two different species [6]. Primary infection, i.e., the first contact with the pathogen usually occurs within the first two years of life [7], though it has also been described to occur in adults [8]. After primary infection, HHV-6 establishes lifelong latency. Periodic reactivations usually have no impact on the health of an immunocompetent host; however, they contribute to the transmission of the virus through saliva [9]. In immunocompetent individuals, primary infections by HHV-6 cause acute febrile illness that can be associated with seizures, skin rash, and other symptoms in the gastrointestinal and respiratory tracts [10]. Roseola infantum (or *exanthema subitum*) is the typical disease manifestation in infants, being caused mainly by HHV-6B [11]. Most of the time, acute infections are uncomplicated but severe clinical manifestations can occur, such as hepatitis, thrombocytopenia, infectious-mononucleosis-like syndrome, gastroenteritis, myocarditis, neurological complications, and meningoencephalitis [12, 13]. In fact, severe manifestations are mainly observed in immunocompromised hosts, with reports of encephalitis and pneumonitis in bone marrow and hematopoietic stem-cell transplant recipients and encephalitis in HIV-infected individuals [14–16]. In the United States (US), Europe and Japan, HHV-6B contributes to the majority of primary infections in infants and young children [17–19]. A study conducted in an emergency department in the US showed that HHV-6 infection was responsible for 20% of the visits due to febrile illnesses among children six-to-12 months old [12, 13].

In this study, we hypothesized that HHV-6 infection was a potential cause of hospital admission for children without a laboratory-confirmed diagnosis when the presence of an initial array of pathogens was assessed. In addition, we aimed to determine the frequencies of HHV-6A and HHV-6B in positive samples.

## Material and methods

### Study population

This is a secondary analysis of a prospective, cross-sectional study that investigated the cause of infections in 600 children ≤ 15 years with fever (rectal or axillary temperature ≥ 38 °C) hospitalized at the Albert Schweitzer Hospital in Lambaréné, Gabon, Central Africa between August 2015 and March 2016. Blood samples were collected once at admission from each participant. In a subset of children, the causative pathogen of fever could not be determined when assessing the presence of a pre-defined panel of pathogens, including *Plasmodium* species, a range of bacteria (e.g. Salmonella, Shigella, and Klebsiella species) and respiratory viruses (e.g. Influenza, Rhinovirus, Respiratory syncytial virus), among others. The complete list of evaluated pathogens can be found in the earlier publication [4]. To investigate if HHV-6 could have been a causative pathogen for hospital admission, we investigated and compared the presence of HHV-6 in three groups of children: (1) the core group of interest (n = 91): a subset of hospitalized children without a diagnosed causing pathogen of this study; (2) a hospitalized control group (n = 91): age-matched children from the same study in whom the potentially disease-causing pathogen had previously been identified; (3) a healthy control group (n = 91): children from a cross-sectional study that collected blood samples from individuals ≥ 12 months to 60 years[20] performed in February/March 2016 in a rural area of Gabon that is neighboring to the study site (Fougamou and surrounding villages).

### HHV-6 detection

DNA was isolated from whole blood samples taken from the participants using a commercial kit and stored at – 20 °C until use. A real-time quantitative PCR (PanHHV-6 qPCR) was performed for the detection of HHV-6 using primers (fwd 5´- GAC AAT CAC ATG CCT GGA TAA TG-3´ and rev 5´-TGT AAG CGT GTG GTA ATG GAC TAA-3´) and probe (5´-FAM AGC AGC TGG CGA AAA GTG CTG TGC TAMRA-3´) targeting a conserved region of the U65–U66 genes of HHV-6 as previously described [21]. Reaction volume was 10 µl containing 1 × Bioline® 2 × SensiFAST™ Probe No-ROX, 200 nM of each primer, 100 nM of probe and 2 µl extracted DNA. Samples were assayed in duplicates in the LightCycler® 480 Instrument II (Roche Diagnostics). All assays included plasmid containing the DNA target sequence and DNA from HHV-6 culture as HHV-6 positive controls, and DNA from a non-infected person and nuclease-free water as negative controls. The quantification cycle (Cq) values were calculated with the second derivative maximum method using the LightCycler® 480 software, version 1.5.1.62.

### HHV-6 species identification

Positive samples by PanHHV-6 qPCR were further assessed by qPCR for HHV-6 species identification using the commercial kits Primerdesign™ Human herpes virus 6, variants A and B Standard Kits (Genesig®, UK) with total reaction volume modified to 10 µl. Positive controls for each species included in all experiments were provided in the kits.

### Statistical analysis

Data analysis was performed using R version 4.2.2. Odds ratios (OR) were calculated from logistic regression model with the healthy control group as reference. Age and sex were added to the model as independent variables, and 95% confidence intervals (CI) calculated.

### Ethical considerations

The study investigating the causes of fever in hospitalized children was approved by the Scientific Review Board and the Institutional Ethics Committee of Centre de Recherches Médicales de Lambaréné (CERMEL), and the study protocol submitted to the Gabon National Ethics Committee on 06 February 2015 and approved (Number 006/2015/SG/P) on 28 February 2015. Written informed consent was obtained from all parents/legal guardians of included children prior to enrolment, as well as assent from children ≥ 8 years.

The cross-sectional study from which samples were used as the healthy control group obtained ethics approval from the responsible ethics committee, the Institutional Ethics Committee of the Centre de Recherches Médicales de Lambaréné (CEI-007/2014). Signed informed consent was obtained from adults ≥ 18 years or the legal guardian in case of minors, and assent was additionally obtained from adolescents ≥ 12 years old.

## Results

In total, 273 children were assessed for HHV-6, 91 in each group. Age ranged from two months to 14 years, and median (IQR) age was 36 (12 – 71) months. Females corresponded to 52% (n = 143) of the population studied.

Overall, HHV-6 DNA was detected in 119/273 (44%) children in our study population (Table 1). No difference in frequency of the diagnosis HHV-6 infection was observed between males and females (Table 2). Logistic regression analysis showed no confounding effect of age (p = 0.0552) and sex (p = 0.743), whereas being part of the group of interest increases the odds of being infected by HHV-6 (p = 3.06e-06). In the group of interest, 58/91 (64%) children were positive for the PanHHV-6 qPCR, as compared to 37/91 (41%) in the hospitalized control group. In the group of interest there were statistically significant differences in the odds of being HHV-6 positive (OR = 4.62, 95% CI [2.46, 8.90]) compared to the hospitalized control group (OR = 1.79, 95% CI [0.95, 3.41]). In the healthy control group only 24/91 (26%) were positive by PanHHV-6 qPCR (Table S1). Taken together, HHV-6 was detected in 52% (95/182) of the hospitalized children.

**Table 1:** PanHHV-6 qPCR results

	Group of interest n = 91	Hospitalized control group n = 91	Healthy control group n = 91	Total
Positive	58 (64%)	37 (41%)	24 (26%)	119 (44%)
Negative	33 (36%)	54 (59%)	67 (74%)	154 (56%)

**Table 2:** Demographic characteristics of individuals positive (pos) for HHV-6 by PanHHV-6 qPCR in the different groups assessed

Group	Group of interest		Hospitalized control		Healthy control		Total	
Total tested, positive	n = 91, pos = 58		n = 91, pos = 37		n = 91, pos = 24		n = 273, pos = 119	
Sex								
Male	32 (55%)		19 (51%)		9 (38%)		60 (50%)	
Female	26 (45%)		18 (49%)		15 (62%)		59 (50%)	
Age, months								
< 24	30 (52%)		21 (57%)		10 (42%)		61 (51%)	
≥ 24	28 (48%)		16 (43%)		14 (58%)		58 (49%)	

Group of interest = hospitalized children without a diagnosed causing pathogen. Hospitalized control = hospitalized children in whom the potentially disease-causing pathogen had previously been identified. Healthy control = healthy control group

**Fig. 1:**
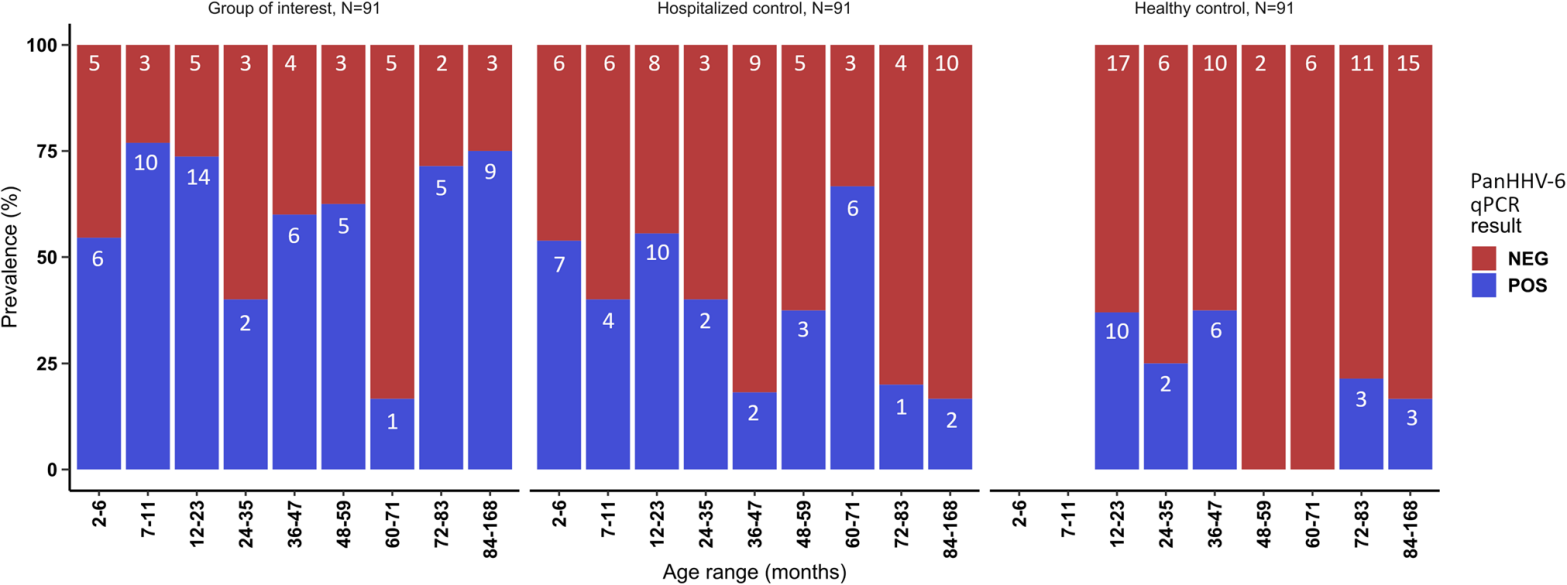
Distribution of HHV-6 DNA detection by PanHHV-6 qPCR in the different age ranges in the three groups evaluated. Absolute numbers are given inside the bars. *NEG* negative, *POS* positive

In total, the proportion of HHV-6 infection in children < 24 months (61/111, 55%) was higher than in older children (58/162, 36%). Figure 1 shows the distribution of HHV-6 among the population studied stratified by age.

All positive samples by PanHHV-6 qPCR (n = 119) were assessed for HHV-6 species using a commercial kit. Species determination was successful in 116/119 (97.5%) samples. Exclusively HHV-6B was detected in all samples.

## Discussion

This study is a secondary analysis of a main study that assessed the causes of fever in hospitalized children in Gabon, Central Africa. HHV-6 DNA was detected in a high proportion of children hospitalized due to febrile illness in whom the cause of fever could not be determined when a predefined panel of pathogens was assessed. In comparison, the prevalence of HHV-6 in a group from the same cohort of hospitalized children in whom the potentially disease-causing pathogen had previously been identified and in a healthy control group of children from the same area were lower, therefore suggesting HHV-6 as the cause of hospitalization for some of the children in the first group.

The results in the hospitalized group also confirm previous analysis by unbiased metagenomic next-generation sequencing [22] that detected HHV-6B in a pool of 20 serum samples collected from children < 5 years from the same cohort. Our results show a detection of HHV-6 DNA comparably high (52.2%, 95/182) in the hospitalized group. In a cohort of patients suspected of yellow fever in the Democratic Republic of Congo, HHV-6 was detected in only 7.8% (15/190) of the group aged 0–15 years [23]. In Senegal, HHV-6 detection was even lower (2.8%, 77/2768) in the pediatric group of measles-suspected patients. Both studies used qPCR for detection of HHV-6 DNA. This discrepancy could be explained by the fact that in our study, only a subset of the hospitalized children was assessed for HHV-6, especially those without a conclusive diagnosis. However, also in our healthy control group, a relatively high proportion of children was positive for HHV-6 DNA, suggesting asymptomatic infection in those. HHV-6 can integrate into the telomeres of host´s chromosome (ciHHV-6) [24] and it is difficult to distinguish this from active viral replication, however, this is usually a rare event and generally occurs in around 1% of the population [25]. A high quantity of HHV-6 DNA in the blood could indicate ciHHV-6 [26] as each host cell with DNA would also carry a copy of viral DNA. However, in our cohort, most of the individuals showed high Cq values in the qPCR, indicating low copies of viral DNA.

Overall, the prevalence of HHV-6 in children < 24 months was higher in comparison with older children. Previous studies showed that the majority of primary infections occurs within the first two years of life [7]. In our study, it is likely that the distribution of HHV-6 infections does not correspond solely to those of primary infections as seen in the US by Hall and colleagues [10] but could be a result of virus reactivation. HHV-6 reactivation usually occurs in immunosuppressed individuals such as recipients of organs transplant and AIDS patients. Although malaria can cause immunosuppression, no association between acute malaria and HHV-6 reactivation was found in children from Uganda [27]. However, it can be the case that an infection by a pathogen other than *Plasmodium* could have played a role in HHV-6 reactivation in some children of our cohort.

In our study population, HHV-6 infections were caused exclusively by HHV-6B regardless of whether they were found in the healthy or hospitalized group. This result is in accordance with other studies that found only HHV-6B in children presenting to pediatric emergency departments in Zambia and the US [28, 29]. In contrast, a study conducted in Zambia [30] found HHV-6A in 48 (86%) of asymptomatic HHV-6 positive samples in a pediatric cohort, whereas HHV-6B was found in only one (2%) sample and mixed infections with HHV-6A and B in seven (13%) out of the 56 samples with genotyping results. Interspecies recombination between HHV-6A and HHV-6B strains showed by whole-genome sequencing [31] could explain these contrasting results. Although the species differentiation has no impact on the management of HHV-6 infection, distinction between the two is crucial for a comprehensive characterization of clinical aspects, epidemiology, etiologic associations, and future species-specific treatment.

Concerning limitations of this study, only a subset of hospitalized children was assessed for detection of HHV-6 DNA (182/600). Therefore, the overall presence of HHV-6 as co-infections in this population could not be determined. The higher proportion of children with a positive HHV-6 PCR in the group of hospitalized children in whom the potentially disease-causing pathogen had previously been identified versus the healthy control group of the cross-sectional study can lead to the assumption that some of these children have been hospitalized because of an HHV-6 infection and not because of the other diagnosed pathogen. To further investigate this hypothesis additional analysis would be necessary, e.g., with follow up samples to analyze conversion of IgM to IgG. However, the absence of follow up samples poses another limitation of this study. In addition, the healthy control group only included children ≥ 12 months and therefore age match did not fit completely with the young children from the group of interest. However, when adding age and sex to the model, the results showed only minimal confounding of these variables.

Overall, in many cases it is difficult to differentiate which pathogen is the one responsible for hospital admission of a patient, as often the combination of pathogens can lead to severe disease. Diagnosis of HHV-6 infection can be done by serological as well as by direct means (usually PCR). If inclusion of HHV-6 in the general diagnostic panel should be considered depends on many different aspects, as especially in low- and middle-income countries, advanced diagnostic tools in a general health-care setting have to be selected based also on the aspect of cost-effectiveness. However, knowledge of the pathogens present enables informed selection of the most important pathogens for the respective region.

## Conclusions

In this study, HHV-6 DNA was detected in 52% of hospitalized children in Gabon, Central Africa, with HHV-6B being exclusively detected in all samples. HHV-6 was most prevalent in the group of children without a diagnosed causing pathogen (64%) compared to hospitalized and healthy control groups, suggesting HHV-6 as a pathogen that may cause hospital admission in young children in Gabon,—as an example for the tropical rain forest area of Central Africa. Although locally relevant diseases such as malaria contribute the majority of pediatric hospitalizations in these countries, our results show the importance of also considering common childhood diseases as HHV-6 when diagnosing febrile illness in children in low- and middle-income countries.

### Supplementary Information

Below is the link to the electronic supplementary material.

Supplementary file1 (DOCX 39 KB)

## Data Availability

Further data of this study are available from the corresponding author upon reasonable request.
