# Supplementary material for: HHV-6 infections in hospitalized young children of Gabon
Source: Infection. 2023 Jul 27;51(6):1759–65. doi: 10.1007/s15010-023-02077-w (PMC10665219; doi:10.1007/s15010-023-02077-w)
Supplement: Supplementary file 1 — Supplementary file1 (DOCX 39 KB) [file 15010_2023_2077_MOESM1_ESM.docx]

Table S1. PanHHV-6 qPCR results of children from Gabon, n = 273.

| **Sample ID** | **Group** | **PanHHV-6 mean Cq** | **PanHHV-6 result** |
| --- | --- | --- | --- |
| 1 | Interest | 32,36 | POS |
| 2 | Interest | 32,45 | POS |
| 3 | Interest | 32,22 | POS |
| 4 | Interest | 32,38 | POS |
| 5 | Interest | 32,51 | POS |
| 6 | Interest | 33,79 | POS |
| 7 | Interest | 33,40 | POS |
| 8 | Interest | 34,88 | POS |
| 9 | Interest | 35,15 | POS |
| 10 | Interest | 32,58 | POS |
| 11 | Interest | 32,23 | POS |
| 12 | Interest | 32,13 | POS |
| 13 | Interest | 32,33 | POS |
| 14 | Interest | 32,47 | POS |
| 15 | Interest | 32,07 | POS |
| 16 | Interest | 32,62 | POS |
| 17 | Interest | 32,83 | POS |
| 18 | Interest | 32,67 | POS |
| 19 | Interest | 32,18 | POS |
| 20 | Interest | 34,50 | POS |
| 21 | Interest | 32,67 | POS |
| 22 | Interest | 32,70 | POS |
| 23 | Interest | 32,73 | POS |
| 24 | Interest | 32,85 | POS |
| 25 | Interest | 34,36 | POS |
| 26 | Interest | 32,35 | POS |
| 27 | Interest | 32,95 | POS |
| 28 | Interest | 32,57 | POS |
| 29 | Interest | 36,32 | POS |
| 30 | Interest | 34,94 | POS |
| 31 | Interest | 32,31 | POS |
| 32 | Interest | 33,79 | POS |
| 33 | Interest | 33,19 | POS |
| 34 | Interest | 34,14 | POS |
| 35 | Interest | - | NEG |
| 36 | Interest | - | NEG |
| 37 | Interest | - | NEG |
| 38 | Interest | - | NEG |
| 39 | Interest | - | NEG |
| 40 | Interest | 34,14 | POS |
| 41 | Interest | - | NEG |
| 42 | Interest | - | NEG |
| 43 | Interest | - | NEG |
| 44 | Interest | 35,04 | POS |
| 45 | Interest | - | NEG |
| 46 | Interest | 34,42 | POS |
| 47 | Interest | 34,01 | POS |
| 48 | Interest | - | NEG |
| 49 | Interest | 33,93 | POS |
| 50 | Interest | 34,89 | POS |
| 51 | Interest | 32,20 | POS |
| 52 | Interest | 34,38 | POS |
| 53 | Interest | 33,05 | POS |
| 54 | Interest | - | NEG |
| 55 | Interest | - | NEG |
| 56 | Interest | 34,08 | POS |
| 57 | Interest | 37,45 | POS |
| 58 | Interest | - | NEG |
| 59 | Interest | - | NEG |
| 60 | Interest | 34,87 | POS |
| 61 | Interest | - | NEG |
| 62 | Interest | - | NEG |
| 63 | Interest | - | NEG |
| 64 | Interest | - | NEG |
| 65 | Interest | - | NEG |
| 66 | Interest | - | NEG |
| 67 | Interest | - | NEG |
| 68 | Interest | 26,98 | POS |
| 69 | Interest | - | NEG |
| 70 | Interest | 35,31 | POS |
| 71 | Interest | - | NEG |
| 72 | Interest | - | NEG |
| 73 | Interest | - | NEG |
| 74 | Interest | 34,61 | POS |
| 75 | Interest | 34,71 | POS |
| 76 | Interest | - | NEG |
| 77 | Interest | - | NEG |
| 78 | Interest | 34,72 | POS |
| 79 | Interest | 32,04 | POS |
| 80 | Interest | 36,49 | POS |
| 81 | Interest | - | NEG |
| 82 | Interest | - | NEG |
| 83 | Interest | 34,54 | POS |
| 84 | Interest | 34,37 | POS |
| 85 | Interest | - | NEG |
| 86 | Interest | 35,01 | POS |
| 87 | Interest | - | NEG |
| 88 | Interest | - | NEG |
| 89 | Interest | 35,61 | POS |
| 90 | Interest | - | NEG |
| 91 | Interest | 38,08 | POS |
| 92 | Hospitalized_control | - | NEG |
| 93 | Hospitalized_control | 36,88 | POS |
| 94 | Hospitalized_control | 34,69 | POS |
| 95 | Hospitalized_control | - | NEG |
| 96 | Hospitalized_control | 35,79 | POS |
| 97 | Hospitalized_control | 32,77 | POS |
| 98 | Hospitalized_control | 34,42 | POS |
| 99 | Hospitalized_control | - | NEG |
| 100 | Hospitalized_control | - | NEG |
| 101 | Hospitalized_control | - | NEG |
| 102 | Hospitalized_control | - | NEG |
| 103 | Hospitalized_control | - | NEG |
| 104 | Hospitalized_control | 34,59 | POS |
| 105 | Hospitalized_control | 31,05 | POS |
| 106 | Hospitalized_control | - | NEG |
| 107 | Hospitalized_control | - | NEG |
| 108 | Hospitalized_control | 32,24 | POS |
| 109 | Hospitalized_control | 32,71 | POS |
| 110 | Hospitalized_control | 35,29 | POS |
| 111 | Hospitalized_control | - | NEG |
| 112 | Hospitalized_control | 32,48 | POS |
| 113 | Hospitalized_control | - | NEG |
| 114 | Hospitalized_control | - | NEG |
| 115 | Hospitalized_control | - | NEG |
| 116 | Hospitalized_control | - | NEG |
| 117 | Hospitalized_control | 32,71 | POS |
| 118 | Hospitalized_control | - | NEG |
| 119 | Hospitalized_control | 30,65 | POS |
| 120 | Hospitalized_control | - | NEG |
| 121 | Hospitalized_control | - | NEG |
| 122 | Hospitalized_control | 35,27 | POS |
| 123 | Hospitalized_control | 34,30 | POS |
| 124 | Hospitalized_control | - | NEG |
| 125 | Hospitalized_control | 34,91 | POS |
| 126 | Hospitalized_control | - | NEG |
| 127 | Hospitalized_control | 29,66 | POS |
| 128 | Hospitalized_control | - | NEG |
| 129 | Hospitalized_control | - | NEG |
| 130 | Hospitalized_control | - | NEG |
| 131 | Hospitalized_control | - | NEG |
| 132 | Hospitalized_control | 34,35 | POS |
| 133 | Hospitalized_control | - | NEG |
| 134 | Hospitalized_control | 35,40 | POS |
| 135 | Hospitalized_control | - | NEG |
| 136 | Hospitalized_control | - | NEG |
| 137 | Hospitalized_control | 35,28 | POS |
| 138 | Hospitalized_control | - | NEG |
| 139 | Hospitalized_control | - | NEG |
| 140 | Hospitalized_control | 32,24 | POS |
| 141 | Hospitalized_control | - | NEG |
| 142 | Hospitalized_control | 35,03 | POS |
| 143 | Hospitalized_control | - | NEG |
| 144 | Hospitalized_control | - | NEG |
| 145 | Hospitalized_control | 35,64 | POS |
| 146 | Hospitalized_control | - | NEG |
| 147 | Hospitalized_control | 32,67 | POS |
| 148 | Hospitalized_control | 34,41 | POS |
| 149 | Hospitalized_control | - | NEG |
| 150 | Hospitalized_control | - | NEG |
| 151 | Hospitalized_control | 31,72 | POS |
| 152 | Hospitalized_control | - | NEG |
| 153 | Hospitalized_control | - | NEG |
| 154 | Hospitalized_control | - | NEG |
| 155 | Hospitalized_control | - | NEG |
| 156 | Hospitalized_control | - | NEG |
| 157 | Hospitalized_control | - | NEG |
| 158 | Hospitalized_control | 34,61 | POS |
| 159 | Hospitalized_control | - | NEG |
| 160 | Hospitalized_control | 35,38 | POS |
| 161 | Hospitalized_control | 35,31 | POS |
| 162 | Hospitalized_control | - | NEG |
| 163 | Hospitalized_control | 28,43 | POS |
| 164 | Hospitalized_control | - | NEG |
| 165 | Hospitalized_control | - | NEG |
| 166 | Hospitalized_control | 35,98 | POS |
| 167 | Hospitalized_control | 36,13 | POS |
| 168 | Hospitalized_control | - | NEG |
| 169 | Hospitalized_control | - | NEG |
| 170 | Hospitalized_control | - | NEG |
| 171 | Hospitalized_control | 34,69 | POS |
| 172 | Hospitalized_control | - | NEG |
| 173 | Hospitalized_control | - | NEG |
| 174 | Hospitalized_control | - | NEG |
| 175 | Hospitalized_control | - | NEG |
| 176 | Hospitalized_control | 35,33 | POS |
| 177 | Hospitalized_control | - | NEG |
| 178 | Hospitalized_control | 33,49 | POS |
| 179 | Hospitalized_control | 34,13 | POS |
| 180 | Hospitalized_control | - | NEG |
| 181 | Hospitalized_control | - | NEG |
| 182 | Hospitalized_control | 35,48 | POS |
| 183 | Healthy_control | - | NEG |
| 184 | Healthy_control | 34,72 | POS |
| 185 | Healthy_control | - | NEG |
| 186 | Healthy_control | 34,69 | POS |
| 187 | Healthy_control | - | NEG |
| 188 | Healthy_control | - | NEG |
| 189 | Healthy_control | - | NEG |
| 190 | Healthy_control | 33,48 | POS |
| 191 | Healthy_control | 34,42 | POS |
| 192 | Healthy_control | 33,32 | POS |
| 193 | Healthy_control | - | NEG |
| 194 | Healthy_control | 35,47 | POS |
| 195 | Healthy_control | - | NEG |
| 196 | Healthy_control | 33,51 | POS |
| 197 | Healthy_control | 34,20 | POS |
| 198 | Healthy_control | - | NEG |
| 199 | Healthy_control | - | NEG |
| 200 | Healthy_control | - | NEG |
| 201 | Healthy_control | - | NEG |
| 202 | Healthy_control | - | NEG |
| 203 | Healthy_control | - | NEG |
| 204 | Healthy_control | 35,45 | POS |
| 205 | Healthy_control | - | NEG |
| 206 | Healthy_control | - | NEG |
| 207 | Healthy_control | - | NEG |
| 208 | Healthy_control | - | NEG |
| 209 | Healthy_control | - | NEG |
| 210 | Healthy_control | - | NEG |
| 211 | Healthy_control | - | NEG |
| 212 | Healthy_control | - | NEG |
| 213 | Healthy_control | - | NEG |
| 214 | Healthy_control | - | NEG |
| 215 | Healthy_control | - | NEG |
| 216 | Healthy_control | - | NEG |
| 217 | Healthy_control | - | NEG |
| 218 | Healthy_control | - | NEG |
| 219 | Healthy_control | - | NEG |
| 220 | Healthy_control | 32,55 | POS |
| 221 | Healthy_control | - | NEG |
| 222 | Healthy_control | - | NEG |
| 223 | Healthy_control | 33,73 | POS |
| 224 | Healthy_control | - | NEG |
| 225 | Healthy_control | 36,43 | POS |
| 226 | Healthy_control | - | NEG |
| 227 | Healthy_control | - | NEG |
| 228 | Healthy_control | - | NEG |
| 229 | Healthy_control | - | NEG |
| 230 | Healthy_control | - | NEG |
| 231 | Healthy_control | - | NEG |
| 232 | Healthy_control | 33,62 | POS |
| 233 | Healthy_control | - | NEG |
| 234 | Healthy_control | 32,96 | POS |
| 235 | Healthy_control | 34,91 | POS |
| 236 | Healthy_control | - | NEG |
| 237 | Healthy_control | - | NEG |
| 238 | Healthy_control | 33,20 | POS |
| 239 | Healthy_control | - | NEG |
| 240 | Healthy_control | - | NEG |
| 241 | Healthy_control | 34,34 | POS |
| 242 | Healthy_control | - | NEG |
| 243 | Healthy_control | - | NEG |
| 244 | Healthy_control | - | NEG |
| 245 | Healthy_control | - | NEG |
| 246 | Healthy_control | - | NEG |
| 247 | Healthy_control | - | NEG |
| 248 | Healthy_control | 35,23 | POS |
| 249 | Healthy_control | - | NEG |
| 250 | Healthy_control | 33,82 | POS |
| 251 | Healthy_control | - | NEG |
| 252 | Healthy_control | - | NEG |
| 253 | Healthy_control | - | NEG |
| 254 | Healthy_control | - | NEG |
| 255 | Healthy_control | 35,42 | POS |
| 256 | Healthy_control | 34,53 | POS |
| 257 | Healthy_control | 35,65 | POS |
| 258 | Healthy_control | - | NEG |
| 259 | Healthy_control | - | NEG |
| 260 | Healthy_control | - | NEG |
| 261 | Healthy_control | - | NEG |
| 262 | Healthy_control | - | NEG |
| 263 | Healthy_control | - | NEG |
| 264 | Healthy_control | 34,43 | POS |
| 265 | Healthy_control | - | NEG |
| 266 | Healthy_control | - | NEG |
| 267 | Healthy_control | - | NEG |
| 268 | Healthy_control | 35,64 | POS |
| 269 | Healthy_control | - | NEG |
| 270 | Healthy_control | - | NEG |
| 271 | Healthy_control | - | NEG |
| 272 | Healthy_control | - | NEG |
| 273 | Healthy_control | - | NEG |

Cq = quantification cycle, POS = positive, NEG = negative.
